# Clinical importance of IL-22 cascade in IBD

**DOI:** 10.1007/s00535-017-1401-7

**Published:** 2017-10-26

**Authors:** Atsushi Mizoguchi, Arisa Yano, Hidetomo Himuro, Yui Ezaki, Takayuki Sadanaga, Emiko Mizoguchi

**Affiliations:** 1 Department of Immunology, Kurume University School of Medicine, Asahi-machi, Kurume, Fukuoka 830-0011 Japan; 2 IBD Center, Kurume University Hospital, Kurume, Japan

**Keywords:** Ahr, Indigo naturalis, IL-22BP, IL-23, Mucus

## Abstract

IL-22 is a relatively new cytokine that is characterized by several unique biological properties. In the intestines, the effect of IL-22 is restricted mainly to non-lymphoid cells such as epithelial cells. Interestingly, the expression pattern and major cellular source of IL-22 have distinct difference between large and small intestines. IL-22 possesses an ability to constitutively activate STAT3 for promoting epithelial cell regeneration and reinforcing mucosal barrier integrity through stimulating the expression of anti-bacterial peptide and mucins. Of note, IL-22 is characterized as a two-faced cytokine that can play not only protective but also deleterious roles in the intestinal inflammation depending on the cytokine environment such as the expression levels of IL-23, T-bet, and IL-22 binding protein. Most importantly, clinical relevance of IL-22 to inflammatory bowel disease has been well highlighted. Mucosal healing, which represents the current therapeutic goal for IBD, can be induced by IL-22. Indeed, indigo naturalis, which can activate IL-22 pathway through Ahr, has been shown in a clinical trial to exhibit a strong therapeutic effect on ulcerative colitis. Despite the beneficial effect of IL-22, continuous activation of the IL-22 pathway increases the risk of colitis-associated cancer, particularly in patients with an extended history of IBD. This review article discusses how IL-22 regulates colitis, how beneficial versus deleterious effects of IL-22 is determined, and why IL-22 represents a promising target for IBD therapy.

## Introduction

Interleukin (IL)-22 is a relatively new cytokine that was discovered in 2000 in both humans and mice [1, 2]. IL-22 was identified in humans as a new ligand of IL-10R2 that can activate STATs 1, 3, and 5 [1] and simultaneously identified in mice as an IL-10-related T cell-inducible factor that was detected in thymic lymphomas stimulated with IL-9 [2]. Rapid progress in the IL-22 biology has since been made in basic and clinical science, both of which have demonstrated its unique properties [3–6]. For example, expression of IL-22 receptor (IL-22R) is restricted to non-hematopoietic cells such as intestinal and respiratory epithelial cells, keratinocytes, and hepatocytes, allowing IL-22 to specifically target innate immune responses without directly affecting adaptive immune cells [7]. Of note, the function of IL-22 has been figuratively called “a sheep in wolf’s clothing” [8] or “a two-headed cytokine” [4] due to its dual abilities to not only exacerbate but also improve inflammatory responses. In addition, IL-22 exhibits unique expression pattern particular in the intestine. IL-22 is constitutively expressed in the small intestine to maintain the epithelial integrity that provides the first line of defense against enteric microorganisms for preserving appropriate host–microbial interactions [9, 10]. In contrast, IL-22 represents an inducible cytokine in the large intestine where IL-22 expression is hardly detectable under healthy state and can be induced under inflammatory conditions such as inflammatory bowel disease (IBD) [11–13]. The clinical relevance of IL-22 to IBD has been well highlighted by the fact that the majority of IL-22-associated molecules are encoded by IBD susceptibility genes [3]. In addition, IL-22 signaling may provide a clue to solve a long-standing epidemiologic mystery in IBD regarding a negative association of cigarette smoking with the development of ulcerative colitis (UC).

### Distinct IL-22 expression pattern in small versus large intestine

There are distinct expression patterns of IL-22 between small and large intestines [3]. IL-22 is constitutively expressed in the small intestine of humans and mice to maintain epithelial barrier integrity against enteric microorganisms that play an important role in the pathogenesis of IBD [9, 10, 14]. In contrast, IL-22 expression, which is rarely detectable in the large intestine (colon) of humans and mice under healthy condition [11–13, 15–17], is induced by a wide variety of inflammatory conditions ranging from IBD to infection [11–13, 15–17]. However, the expression level of inducible IL-22 may differ depending on the types of inflammation. For example, the expression level of IL-22 in the inflamed colon is higher in Crohn’s disease (CD) patients as compared to UC patients [11, 12, 15]. Similarly, much higher expression level of IL-22 has been reported in the inflamed colon of a mouse model of CD (CD45RB model) as compared to a mouse model of UC (TCRα KO mice) [12]. CD is characterized by dominant Th1/Th17 responses, whereas UC is associated with enhanced Th2 response [18]. In addition, atopic dermatitis, which exhibits a dominant Th2 response, is associated with lower levels of IL-22 expressions as compared to psoriasis characterized by enhanced Th1/Th17 responses [19]. Therefore, it is possible that IL-22 expression, which is elicited by inflammatory insults, is further conditioned by the local cytokine environments (e.g., Th1 versus Th2).

### Cellular sources of IL-22 in the intestine

IL-22 can be expressed by many cell types, including Th17 CD4^+^ T cells that express both IL-17 and IL-22, Th22 CD4^+^ T cells that express IL-22 without producing IL-17, CD8^+^ T cells, TCRγδ T cells, dendritic cells (DCs), neutrophils, NK cells, and group3 innate lymphoid cell (ILC) population including ILC3 and lymphoid tissue inducer (LTi) cells [4–6, 20]. The major cellular source of IL-22 in the small intestine is RAR-related orphan receptor (ROR)γt-dependent ILC3 that can develop in the absence of enteric bacteria as indicated by the development of ILC3 in germ-free mice [14, 21]. In *Toxoplasma gondii* infection, the major source of IL-22 in the small intestine shifts from ILC3 to CD4^+^ T cells [22].

In human colon, different types of cells may be responsible for the inflammation-induced production of IL-22 depending on the type of inflammation and presumably on the disease stages. The ability of NK cells and Th17 T cells to produce IL-22 was initially reported in the inflamed colon of UC patients [16], and subsequent studies have suggested that IL-22-producing NKp46^+^ NKp44^−^ NK cells more increase in the inflamed colon of CD patients as compared to that of UC patients [23]. A recent study has found an additional cell type that can be responsible for the production of IL-22 in IBD. CD177^+^ neutrophils produce IL-22 for improving colitis and this neutrophil subset increases in the peripheral blood and inflamed intestine of UC and CD patients [20]. In CD patients, CD4^+^ T cells characterized by the expression of natural killer group 2 member D (NKG2D) have been reported to produce IL-22 [24]. Group 3 ILC population is further classified into ILC3 that produces IL-22 and expresses CD56 and LTi cells that produce both IL-17 and IL-22 without expression of CD56 [25]. An increases of both IL-22-producing CD56^+^ ILC3 and IL-17-producing CD56^−^ LTi cells are observed in the inflamed colon of CD patients as compared to UC patients [26]. Alternatively, ILC3 has recently been shown to possess an additional function to serve as immune suppressive antigen presenting cells capable of eliminating enteric bacteria-specific colitogenic CD4^+^ T cells through an MHC class II-dependent manner [27]. In the inflamed colon of pediatric CD patients, the total frequency of ILC3 cells is not altered, but their expression level of MHC class II is reduced [28].

Similar to IBD patients, cellular sources of IL-22 vary in the inflamed colon of mouse IBD models depending on the fundamental disease mechanisms. In a mouse model of CD (CD45RB transfer model), both NK cells and Th17 cells are primarily responsible for the production of IL-22 in the inflamed colon [13]. Alternatively, dendritic cells (DCs) [17], neutrophils [29], and/or TCRγδ T cells [30] represent the major source of IL-22 in an acute colonic injury model, which is induced by oral administration of dextran sulfate sodium (DSS). DCs have also been suggested as a major source of IL-22 in *C. rodentium*- infectious colitis model [31]. In contrast, a study suggested in the *C. rodentium* infectious model that RORγt^+^ LTi cells, but not DCs, produce IL-22 in the inflamed colon [32]. In addition, IL-23-dependent LTi cells, which are characterized by the localization within the intraepithelial compartment of colon, have been proposed to represent a dominant source of colonic IL-22 in the context of *C. rodentium* infection [33]. Taken together, these findings indicate that IL-22 can be produced by many cell types depending on the tissue involved and on the healthy versus disease states.

### Epithelial cell-specific STAT3 activation by IL-22

STAT3 is one of the major signaling molecules involved in the pathogenesis of IBD. However, STAT3 possesses dual abilities to not only improve but also exacerbate intestinal inflammation. Activation of STAT3 in adaptive immune cells such as CD4^+^ T cells contributes for the development of colitis in a mouse model of CD [34]. On the other hand, activation of STAT3 in innate immune cells such as macrophages contributes to the suppression of colitis as indicated by the spontaneous development of colitis in a conditional knockout mouse strain in which the *stat3* gene is specifically deleted in macrophages/neutrophils [35]. In addition, absence of STAT3 in epithelial cells makes mice more susceptible to DSS-induced acute colonic injury [17]. Therefore, to pay careful attention would be needed when STAT3 is considered as a target for the treatment of IBD.

IL-22 activates (phosphorylates) STAT3, to a lesser extent STAT1, and in certain cells STAT5 [36, 37]. This STAT3 activation is mediated by tyrosine kinase 2 (Tyk2) [38]. In addition, IL-22 has been reported to activate Erk1/2, JNK, and p38 MAP in some, not all, epithelial cell lines. A high dose of IL-22 activates Erk1/2, JNK, and p38 MAP kinase in a rat hepatoma cell line [36, 37] and human colonic cancer cell line HT29 [15]. In contrast, inability of IL-22 to activate Erk/1/2 has been shown in a human hepatoma cell line HepG2 [39] and primary epithelial cells isolated freshly from human and mouse colons [12]. IL-22 binds to a heterodimeric receptor composed of IL-22R1 and IL-10R2 [4–6]. IL-10R2 is ubiquitously expressed by majority of cell types, while the expression of IL-22R1 is restricted to non-hematopoietic cells such as epithelial cells, hepatocytes, and keratinocytes [4–6]. This expression pattern of IL-22R1 allows IL-22 to activate STAT3 specifically in intestinal epithelial cells [7]. Indeed, epithelial STAT3 activation induced by intestinal damage has been demonstrated to depend more on IL-22 rather than IL-6, a well-known activator of STAT3 [17]. This strong activation of STAT3 by IL-22 may be explained by the ability of IL-22R1 to constitutively, not transiently, activate STAT3 through constitutive interaction of its C-terminal tail with the coiled-coil domain of STAT3 [40].

### Production of anti-bacterial peptides by IL-22

Dysregulated host–microbial interactions are closely involved in the pathogenesis of IBD. Epithelial cells provide the first barrier against enteric microorganisms, and the ability of IL-22 to reinforce the epithelial barrier integrity has been well documented [3]. IL-22 stimulates the production of different kinds of anti-bacterial peptides such as S100A7, S100A8, S100A9 β-defensin 2, and regenerating gene (Reg) IIIγ and RegIIIβ [17, 31, 41–43]. One of well-studied anti-bacterial peptides in the field of IL-22 may be RegIII family. Bactericidal ability of RegIIIγ against Gram-positive bacteria [44] was first discovered in 2006, and a subsequent study using RegIIIα identified that the bactericidal effect is mediated by the formation of a hexameric membrane-permeabilizing oligomeric pore on Gram-positive bacteria and the bactericidal effect is inhibited by Gram-negative bacteria-derived lipopolysaccharide [45]. Alternatively, a protective role of RegIIIγ and RegIIIβ against Gram-negative bacteria *C. rodentium* has also been proposed [31]. There still remains an issue under debate regarding whether RegIII expression is mediated directly by IL-22-dependent STAT3 activation or not. Several investigators suggested the induction of RegIII γ and β expressions through IL-22-dependent STAT3 activation [17], whereas others suggested the NFκB1-dependent RegIIIγ expression through IL-23 or MyD88 [46, 47]. A recent study has brought an attractive new concept that RegIIIβ represents the initiation, rather than downstream, molecule in IL-22 pathway. RegIIIβ is, at first, produced by epithelial cells through IL-23 receptor signaling, and the epithelial-derived RegIIIβ then recruits IL-22-producing neutrophils that contribute for the improvement of colitis [48].

Other IL-22-associated anti-bacterial peptides that have clinical relevance may be S100A8 and S100A9. S100A8 and S100A9 form a heterodimer complex termed calprotectin, and fecal calprotectin has been widely used as a biomarker for IBD [49]. Although calprotectin is produced mainly by neutrophils, inflammation can induce the ectopic expression of calprotectin in colonic epithelial cells by IL-22 [42]. In addition, expressions of S100A8 and S100A9 are mediated by STAT3 activation [50]. Therefore, it is possible that the fecal calprotectin may reflect not only neutrophil-derived calprotectin but also ectopically expressed calprotectin in epithelial cells by inflammation-induced IL-22.

### Reinforcement of mucus barrier integrity by IL-22

Mucus, which covers the intestinal surface, also plays a major role in preserving epithelial barrier integrity [51]. The mucus layer in the colon is composed of outer and inner stratum; the outer is a loose gel layer where it is unattached to the epithelial surface and provides a place for colonized bacteria, whereas the inner stratum is a firm mucus layer that is directly attached to the epithelial surface to prevent bacterial intrusion [52]. Mucin (Muc) 1, which is a membrane-bound mucin, represents one of the major components in the intestinal mucus [51]. A protective role of Muc1 in mouse models of UC and CD has been demonstrated [53]. The ability of IL-22 to promote the production of functional Muc1 through activation of STAT3 has been demonstrated using human colonic cancer cell lines (T84 and HT29) and primary colonic epithelial cells from mice [12, 54]. Consistent with these findings, STAT3 has been demonstrated to bind within the promoter region of the *muc1* gene [55]. These findings from in vitro and mouse studies are further supported by data from IBD patients. An early onset IBD patient who has no functional IL-10R2 (a receptor of IL-22) due to polymorphism within *il10rb* gene has been reported to lack Muc1 expression [56]. Importantly, Muc1 is a heavily glycosylated protein with over 80% (per total molecular weight) of carbohydrates that are required to form a viscous firm barrier. Therefore, not only the expression of Muc1 but also its complete glycosylation are required to preserve epithelial barrier integrity. Indeed, overexpression of hypoglycosylated Muc1 makes breaches in the mucus barrier [57] and exacerbates colitis [58]. Of note, IL-22 has been demonstrated to promote glycosylation [43, 59]. Therefore, it is possible that the dual abilities to stimulate not only Muc1 expression but also glycosylation allow IL-22 to produce completely firm inner mucus layer to prevent bacterial invasion.

### Mucosal healing by IL-22

IL-22-dependent activation of STAT3 enhances the transcription of anti-apoptotic and pro-proliferative genes such as *birc5, pla2g5, smo, myc*, *mcl1*and *regIα* [16, 60]. The expression of deleted in malignant brain tumor 1 (DMBT1), which may play a role in epithelial cell differentiation, is also induced by IL-22 [61]). These abilities allow IL-22 to promote mucosal healing by stimulating epithelial cell regeneration with goblet cell restitution [12, 17]. In addition, recent studies have demonstrated that the epithelial cell regeneration is initiated by activation of intestinal stem cells through IL-22-dependent activation of STAT3 but not STAT1 [62, 63].

Consistent with these basic observations, the ability of IL-22 to promote mucosal healing has been well documented in vivo by using different experimental approaches. Mucosal healing after epithelial damage induced by DSS was impaired in IL-22-deficient mice [17], IL-23R-deficient mice that lack IL-22 expressions [47], and WT mice treated with neutralizing anti-IL-22 Abs [12]. In addition, a gene therapy to enhance colonic IL-22 expression [12] and an antibody-based targeted delivery of IL-22 to inflamed area [64] promoted the mucosal healing. The mucosal healing was also enhanced by the treatment with Ficz, which is a ligand of Ahr capable of inducing the expression of IL-22 [65]. Since the induction and maintenance of mucosal healing is the major therapeutic goal in the management of IBD [66], the ability to promote mucosal healing clearly highlights the relevance of IL-22 for IBD therapy.

### Newly identified functions of IL-22

Attractive new functions of IL-22 to induce diarrhea for the clearance of enteric pathogens, to dampen adaptive immune responses against enteric bacterial antigens, and to improve endoplasmic reticulum (ER) stress have recently been demonstrated. IL-22 stimulated the expression of a tight junction molecule Claudin-2, which induces water efflux (diarrhea) for the clearance of enteric pathogens [67]. In addition, IL-22 suppressed the antigen uptake of follicular associated epithelium (FAE) that covers Peyer’s and colonic patches where they provide a place for the induction of adaptive immune responses against enteric antigens [68]. Furthermore, IL-22 was shown to reduce epithelial ER/oxidative stress induced by high-fat diets [69].

### Dual roles of IL-22 in intestinal inflammation

As referred to as “a sheep in wolf’s clothing” [8] or “a two-headed cytokine” [4], not only protective but also deleterious roles of IL-22 have been reported in intestinal inflammation models [3]. In infectious models, IL-22-deficient mice are highly susceptible to intestinal inflammation caused by *Citrobacter rodentium* [14, 31, 33, 70], *Salmonella enterica* [71], and candidiasis [72]. In contrast, *Toxoplasma gondii*-induced immunopathology was improved in the absence of IL-22 [22].

Although the involvement of IL-22 in mucosal healing has been reported reproducibly by many groups, the different roles of IL-22 have been demonstrated in chronic colitis models. In a Th2-mediated chronic colitis model (TCRα KO mice) representing UC, IL-22 was shown to play a protective role by reinforcing intestinal mucus barrier function [12]. In a Th1-mediated CD model (CD45RB model) that is induced by the adoptive transfer of naïve CD4^+^ T cells into a immune-deficient host, both T cell-derived and NK cell-derived IL-22 contributed for the suppression of colitis [13]. Ficz, which is a ligand of Ahr capable of inducing IL-22 expression, was reported to improve the colitis of the CD45RB model as well as acute CD model induced by intrarectal administration of trinitrobenzene sulfonic acid (TNBS) [65]. Alternatively, in a chronic colitis model that is induced by the adoptive transfer of memory CD4^+^ T cells, a proinflammatory role of memory T cell-derived IL-22 was proposed [73]. Similarly, IL-23R-dependent IL-22 production caused intestinal inflammation in an innate colitis model that was induced in immune-deficient mice by administration of anti-CD40 mAbs [74].

### Control of IL-22 functions by IL-23R and T-bet

**Fig. 1:**
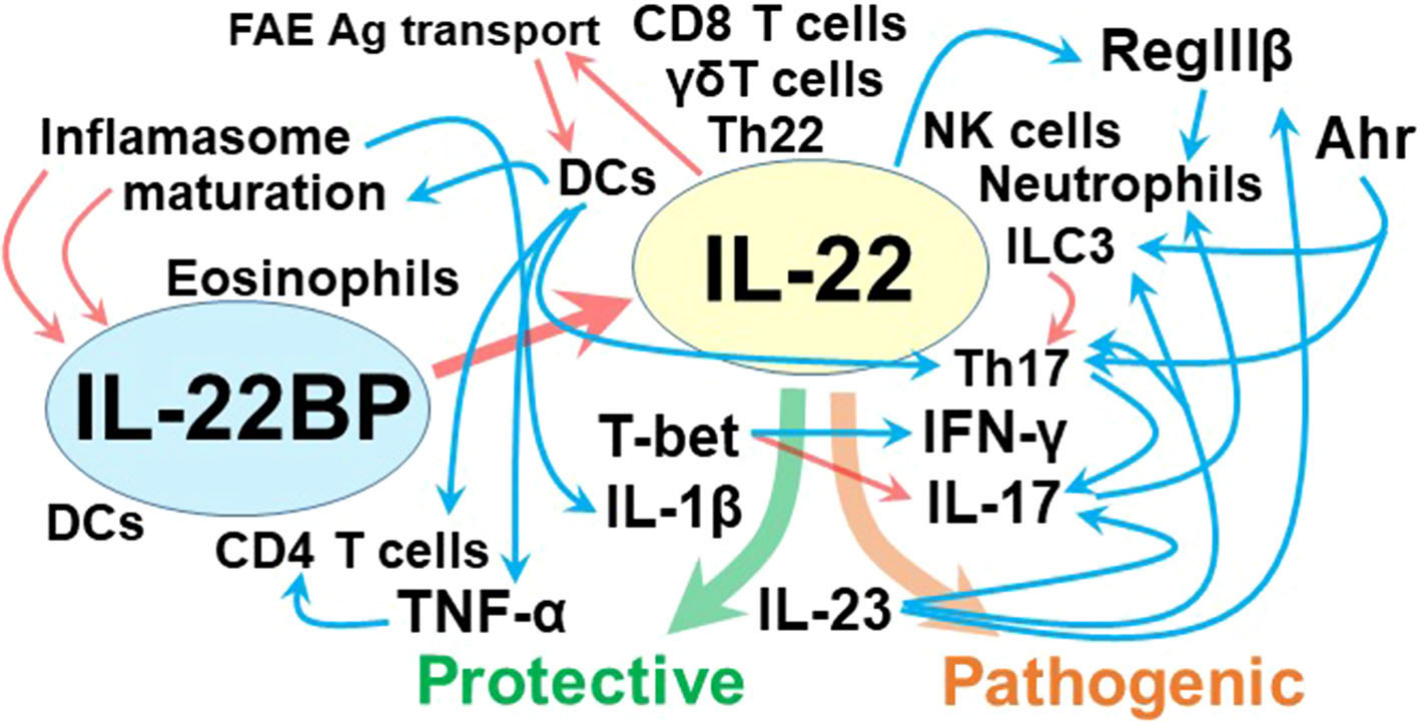
Regulation of IL-22 functions: IL-22 can be produced by many cell types. The production of IL-22 is stimulated by Ahr, and RegIIIβ may have the ability to recruit IL-22-producing neutrophils. IL-22 then plays not only protective but also deleterious roles in intestinal inflammation depending on the cytokine environments. IL-1β and IL-23-medicated activation of epithelial cells may elicit the beneficial effect of IL-22, whereas hyper response of T cells to IL-23 for acquiring their ability to produce both IL-17 and IFN-γ may elicit the deleterious function of IL-22. The activity of IL-22 can be suppressed by an endogenous inhibitor IL-22BP that is produced by DCs, eosinophils, and CD4^+^ T cells. TNF-α stimulates the expression of IL-22BP in CD4^+^ T cells. Alternatively, formation of inflammasome and maturation of DCc may contribute for reducing the IL-22BP expression

It’s becoming increasingly apparent that the function of IL-22 is positively and negatively regulated by the complicated interactions with other factors (Fig. 1). One of the nice examples may be psoriasis, in which the pathogenic potential of IL-22 is elicited only in the presence of IL-17 and IFN-γ [75]. Similarly, IL-22 plays a pro-inflammatory role in the presence of IL-17A and conversely plays a protective role in the absence of IL-17A in bleomycin-induced airway inflammation [76]. In an IBD model, IL-22 plays a pathogenic role under a condition where T cells are hyper responsive to IL-23 for their Th17 differentiation due to absence of T-bet (a transcription factor for Th1 differentiation) [77]. Alternatively, early induction of IL-23 and IL-1β, although both are pro-inflammatory cytokines, are required for initiating the IL-22-dependent tissue repair [78]. In addition, IL-23 signaling in epithelial cells licenses the protective effect of IL-22 on intestinal inflammation. IL-23R-mediated signaling stimulates epithelial cells to produce RegIIIβ, which then recruits IL-22-producing neutrophils capable of improving colitis [48]. Interestingly, an autocrine regulation of IL-22 pathway has also been proposed recently. The RegIIIβ produced by epithelial cells, in turn, inhibits their STAT3 activation in response to IL-22 [79].

### An endogenous inhibitor of IL-22

Activation of IL-22 is tightly and directly regulated by an endogenous inhibitor. IL-22 binds to not only a heterodimeric receptor composed of IL-10R2 and IL-22R1 but also a soluble class II cytokine receptor designated IL-22Ra2 (also called IL-22 binding protein, IL-22BP, or CRF2-10). In comparison with IL-22R1, IL-22BP has 20–1000 fold higher affinity to IL-22 [80–82]. Therefore, IL-22BP can strongly inhibit the biological activity of IL-22 in vitro and in vivo by competing with IL-22R1/IL-22 interaction [12, 80–82]. IL-22BP is highly expressed in placenta, spleen, skin, and lung, and to a lesser extent in large and small intestine of humans [80]. In mouse intestine, DCs [83], CD4^+^ T cells [84], and epithelial cells [85] have been proposed as a producer of IL-22BP. The IL-22BP is highly expressed in the normal colon and significantly reduced during acute inflammation induced by DSS [12, 83, 86]. The inflammation-induced reduction of IL-22BP expression is caused by a formation of inflammasomes that function as a sensor of stress to activate IL-1 and IL-18 [83] or by the maturation of DCs, suggesting immature DCs as a source of IL-22BP [86]. In contrast to mice, rather increased expression of intestinal IL-22BP was observed in patients with IBD, and CD4^+^ T cells [84] and eosinophils [87] have been proposed as the cellular source of IL-22BP. However, more caution may be necessary to evaluate the expression of functional IL-22BP, because there are three functionally different isoforms of IL-22BP in humans but not mice [88]. Isoform 1, which is composed of six exons, is non-functional due to the lack of secretion. Isoform 2, lacking exon 3, represents a homologue of mouse IL-22BP and possesses a strong inhibitory effect on IL-22 activity. Isoform 3, which lacks exons 3 and 5 and partially exon 6, is most abundantly distributed, but the affinity of isoform 3 to IL-22 is 27-folds lower than that of isoform 2 [88].

Since IL-22BP expression became rarely detectable during the acute inflammatory response induced by DSS [12, 83], deficiency of IL-22BP in mice had no significant alteration on the acute inflammation [83]. Alternatively, supplementation of IL-22BP expression delayed the recovery from this acute inflammation by inhibiting IL-22 activity [12]. In a chronic colitis model, TNF-α stimulated the IL-22BP expression in CD4^+^ T cells, leading to the suppression of IL-22-dependent mucosal healing [84]. Indeed, anti-TNF-α treatment reduced the expression of IL-22BP in CD4^+^ T cells of IBD patients [84]. Therefore, this finding may provide a clue to further understand the therapeutic mechanism of anti- TNF-α therapy.

### IL-22-associated IBD susceptibility gene network

Several facts have highlighted the clinical significance of IL-22 to IBD. One of the examples is that the majority of IL-22-associated molecules are encoded by IBD susceptibility genes. IL-23 is a heterodimeric cytokine composed of p40 and p19 subunits. The interaction of IL-23 with IL-23R has been demonstrated to not only stimulate IL-22 expression [5, 6], but also positively and negatively control the function of IL-22 [77, 78]. *Il23r* gene represents one of the major IBD susceptibility genes and the polymorphism was negatively associated with the development of both CD and UC [89, 90]. In addition, *p40* polymorphism was also associated with the development of CD [91].

The receptor of IL-22 is composed of IL-10Rβ and IL-22R1 subunits. Positive association of *il10rb* polymorphisms with CD and UC particularly with early onset of IBD has been well documented [92]. In addition, the *il22* gene is located within a UC-risk locus on chromosome 12q15 [93], and *il22* polymorphism in UC patients has been reported in the Chinese [94], but not Mexican [95], population. The ligation of IL-22 receptor is well known to induce the activation of STAT3 through JAK2 and TYK2. The *stat3*, *jak2*, and *tyk2* all represent well-defined susceptibility genes for CD and to lesser extent UC [89, 90]. The STAT3 activation then stimulates the colonic epithelial cells to produce Muc1 and Muc13 for barrier reinforcement [11, 12, 55]. The *muc13* gene is an IBD susceptibility gene, and the *muc1* gene has been proposed as a candidate susceptible gene of CD [96]. In addition, the ability of IL-22 to directly or indirectly promote the expressions of *fut2, sec1, bcl2115, ptpn22, prdm1, xbp1, nupr1, erbp3, efemp2, chac1, pbbp, cxcl5*, and *sk9a3* has recently been demonstrated by a study using IL-22Ra1-deficient mice [43]. Importantly, all of them have been reported as IBD susceptibility genes. Taken together, these findings highlight the close involvement of IL-22 in the molecular network associated with IBD susceptibility genes.

### A clue to resolve an etiological mystery in UC

A negative association of cigarette smoking with the development UC has been well documented [97]. However, the mechanism is a long-standing question, thus considered as one of ten remaining mysteries in IBD [98]. Aryl hydrocarbon receptor (Ahr), which serves as a sensor that can be activated by a large variety of environmental toxins, can induce the production of IL-22 [99]. Indeed, like IL-22, the beneficial effect of Ahr activation on experimental colitis has been demonstrated [65]. Cigarette smoke contains more than 150 toxins, including dioxin such as 2,3,7,8-tetrachlorodibenzo-*p*-doxin (TCDD) and environmental pollutants such as polycyclic aryl hydrocarbons, of which benzo [α] pyrene (BaP) is a prototype [100]. Of note, TCDD and BaP can activate the Ahr [100], and induction of IL-22 expression by cigarette smoke has recently been demonstrated [101]. These findings may raise an attractive hypothesis that the preventive effect of smoking on UC development is mediated by Ahr-dependent activation of IL-22 pathway.

### Therapeutic application of IL-22

*Lactobacilli* species metabolize tryptophan to indole-3-acetic acid that can activate Ahr for stimulating IL-22 production [102]. Therapeutic benefit of tryptophan on experimental colitis through activation of the IL-22 pathway has also been demonstrated [102, 103]. In addition, the fecal from UC and CD patients was characterized by a low level of tryptophan and indole-3-acetic acid [103].

Xilei-San is a Chinese herbal medicine that has been used for the treatment of UC [104]. Xilei-San contains a high concentration of indigo naturalis, a dye extracted from plants. A recent open-label, prospective pilot study on 20 patients with moderate UC showed that oral administration of indigo naturalis induced mucosal healing in 61% of patients at 8 weeks [105]. Importantly, indigo naturalis is one of the ligands of Ahr, and a recent study in mice demonstrates that the therapeutic effect of indigo naturalis is mediated by Ahr-dependent activation of IL-22 pathway [106]. In addition, the therapeutic potential of other Ahr ligands such as Ficz [65] and ABX464 (the first-in-class anti-HIV drug candidate) [107] against experimental colitis has been reported. These findings clearly highlight the activation of IL-22 as a promising therapeutic measure for IBD, particularly UC. However, more caution may be needed when supplementation of IL-22 is considered for IBD therapy. Chronic inflammation predisposes IBD patients to the risk of colitis-associated cancer that develops through a complex multi-step and multi-factorial processes termed “inflammation-dysplasia-carcinoma sequence” [108]. Hyper activation of STAT3 is one of factors involved in the development of CAC [109], and continuous activation of IL-22 due to lack of IL-22BP has been shown to increase the susceptibility to CAC induced by chronic intestinal inflammation plus a carcinogen AOM [83]. These findings suggest that IL-22/IL-22BP axis critically regulates mucosal healing and tumorigenesis.

## Conclusions

**Fig. 2:**
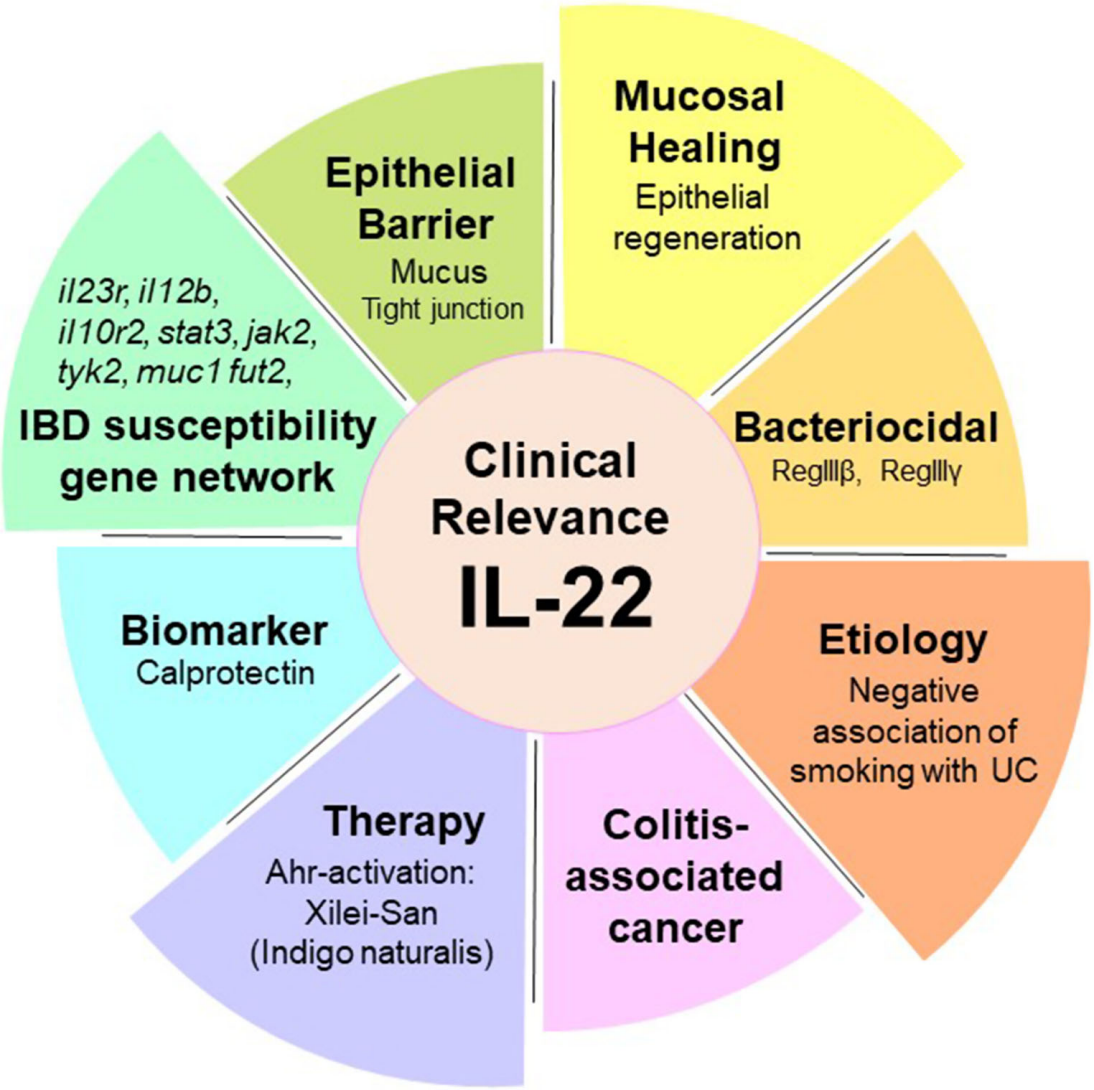
Clinical importance of IL-22 to IBD particularly UC: The clinical relevance of IL-22 can be highlighted by much basic and clinical evidence. For example, IL-22 can promote the mucosal healing in mice, which is a current major goal of IBD therapy. The majority of molecules induced by IL-22 are encoded by IBD susceptibility genes. In addition, IL-22 stimulates the production of calprotectin, which is a useful biomarker of IBD. Interestingly, the IL-22 pathway may provide a clue to resolve a long-standing etiological mystery that cigarette smoking is negatively associated with the development of UC and to further understand the carcinogenesis pathway of colitis-associated cancer. Importantly, a recent pilot study demonstrated the therapeutic effect of Ahr-activation capable of stimulating IL-22 production of mild-to-moderate UC

IL-22 is characterized by numerous clinical relevance to IBD (Fig. 2), including the ability to induce the mucosal healing that represents the current therapeutic goal of IBD, the close involvement in IBD susceptibility gene networks, and the association with CAC, the potential role in an etiological mystery of UC, and the stimulation of expression of a biomarker for IBD. In addition, the IL-22 pathway seems to mediate the therapeutic effect of indigo naturalis and partially of anti-TNFα therapy, both of which have been used for the treatment of IBD. Although much more extensive works still need to fully dissect out the mechanism that determines the beneficial versus deleterious effect of IL-22 on colitis and that stimulates the tumorigenesis pathway, IL-22 activation would be considered to be a promising therapeutic measures for IBD.

## Abbreviations

Ahr: Aryl hydrocarbon receptor
CAC: Colitis-associated cancer
DC: Dendritic cells
DSS: Dextran sulfate sodium
IBD: Inflammatory bowel disease
IL-22BP: IL-22 binding protein
ILC: Innate lymphoid cells
Muc: Mucin
Reg: Regenerating gene
RORγt: RAR-related orphan receptor γt
STAT: Signal transducers and activator of transcription
TCR: T cell receptor
UC: Ulcerative colitis
